# Positive Effect of Breastfeeding on Child Development, Anxiety, and Postpartum Depression

**DOI:** 10.3390/ijerph17082725

**Published:** 2020-04-15

**Authors:** Štefica Mikšić, Boran Uglešić, Jelena Jakab, Dubravka Holik, Andrea Milostić Srb, Dunja Degmečić

**Affiliations:** 1 Faculty of Dental Medicine and Health, Josip Juraj Strossmayer University of Osijek, Crkvena 21, 31 000 Osijek, Croatia; stefica.miksic@fdmz.hr (Š.M.); dubravka.holik@fdmz.hr (D.H.); milostic.andrea@gmail.com (A.M.S.); 2 School of medicine, University of Split, Šoltanska 2, 21000 Split, Croatia; boran-uglesic@mefst.hr; 3 University Hospital Split, Spinčićeva 1, 21000 Split, Croatia; 4 Faculty of Medicine, Josip Juraj Strossmayer University of Osijek, Josipa Huttlera 4, 31 000 Osijek, Croatia; ddegmecic@mefos.hr; 5 University hospital Osijek, Josipa Huttlera 4, 31 000 Osijek, Croatia

**Keywords:** anxiety, breastfeeding, postpartum depression, child development

## Abstract

Background: Postpartum depression is a psychiatric disorder that starts from the second to the sixth week after birth. Breastfeeding is considered a protective factor for postpartum mood swings. This paper aims to examine the effect of breastfeeding on postpartum depression and anxiety, and how it affects child development. Methods: The study included 209 pregnant women, 197 puerperea, and 160 women at the end of the third month after delivery, followed through three time-points. The instruments used in the study were the Edinburgh Postpartum Depression Scale (EPDS), Beck’s Depression Inventory (BDI), and Beck’s Anxiety Inventory (BAI). Results: Postpartum mothers with low risk of PPD breastfed their children more often than mothers with a mild or severe risk of perinatal depression. Mean values on the BDI scale three months after giving birth were higher in mothers who did not breastfeed their child (M = 3.53) than those who did breastfeed their child (M = 2.28). Postpartum anxiety measured by BAI was statistically negatively correlated (rs-, 430) with the duration of breastfeeding. Conclusion: Nonbreastfeeding mothers are more depressed and anxious compared to breastfeeding mothers.

## 1. Introduction

The postpartum period exposes the mother to various demands. This period is considered a potentially vulnerable time for maternal mental health [1]. Mothers face the physical demands of recovering from childbirth, feeling tired and uneasy. Most of the time, mothers are able to take care of their infant and adapt to a new role within the family [2].

At birth, many women suffer a milder mood disorder called “postpartum sadness.” Two days after birth, anxiety symptoms, mood swings and problems with sleep and concentration may occur [3]. However, nearly 15% of postpartum women develop a psychiatric disorder called postpartum depression (PPD) within the first 12 months [4]. PPD usually starts from the second to the sixth week after birth [5]. In women with postpartum sadness (“blues”), symptoms are not very pronounced and usually disappear within two weeks, while in women with PPD, the symptoms are very pronounced and last much longer [6]. Symptoms of PPD include feelings of powerlessness, loneliness, lack of love for the child, a loss of confidence, and loss of interest in daily activities [7,8]. Mothers may also feel anxiety, anger, guilt, and an inability to care for their child, considering themselves to be bad mothers [7,8,9,10], or fear that others will perceive them as inadequate mothers [11]. If untreated, it can have long-lasting effects on both the mother and child [12]. PPD is associated with an increased risk of health problems in children, such as asthma, diabetes, diarrhea, and disrupted neonatal sleep cycle, and may be associated with structural brain abnormalities [13]. Longer episodes of PPD may be associated with psychiatric disorders in children, such as attention deficit hyperactivity disorder (ADHD) and anxiety disorder. The highest risk of PPD is for patients who have already had depressive symptoms during their lifetime. Therefore, antidepressants are proposed for prevention, and psychotherapy is suggested as an alternative approach for patients who do not respond well to therapy [12].

Postpartum anxiety disorders (PPAD) are diagnosed in 4%–39% of pregnant women and 16% of women in the postpartum period [14]. PPAD increase the risk of postpartum depression and have been associated with maternal low self-confidence, low self-efficacy in the parenting role, agitation, overcaring for the child, extreme lability, sleep disturbance, difficulties in concentration, memory loss, and decreased coping capability. Also, women with PPAD breastfeed for shorter periods of time and are less likely to breastfeed exclusively. In the offspring, PPAD has been linked to early complications (insecure attachment behavior, delayed cognitive development, negative temperament), and later adverse child development (low social engagement) [15].

Breastfeeding provides many health benefits for the mother and her child. Breast milk is the best food for a newborn baby, containing all the nutrients they need for healthy growth and development. Breastfeeding promotes craniomandibular development as a result of the sucking reflex, prevents the possibility of developing food allergies, and promotes the child’s neurological development. Breastfed children are at lower risk for diabetes mellitus types 1 and 2, obesity, hypertension, and heart disease later in life [12]. The amount and composition of breast milk changes during lactation, especially in the first month of life, to meet the baby’s needs [16]. In addition to the nutritional importance of breast milk, breastfeeding is a special psychophysical stimulant for mother and baby. During breastfeeding, the mother gives the baby a feeling of warmth, safety, and protection. Establishing warm and secure emotional attachments in childhood is seen as the prevention of various later undesirable behaviors in a child, such as delinquencies, various addictions, violence, and alienation [17].

Studies have shown that breastfeeding is a protective factor in the case of postpartum mood swings [18]. Breastfeeding women develop PPD less frequently, suggesting that some hormonal effects associated with lactation may have protective effects on mental health, such as the antidepressant and anxiolytic effects of oxytocin and prolactin. Lactation has also been associated with attenuated neuroendocrine stress responses, protecting maternal psychological health. Breastfeeding improves maternal emotional involvement and mother-child bonding and interaction [19]. Therefore, it is important to investigate the effect of breastfeeding on postpartum depression and anxiety, and how it affects the development of the child. This study aimed to determine the influence of breastfeeding on PPD and anxiety among women in Đakovština area in Eastern Croatia.

## 2. Materials and Methods

### 2.1. Participants

This prospective cohort epidemiological study [20] was conducted at the Đakovo Health Center in obstetrics/gynecological clinics and during nurse home visits. Participants were randomly selected among pregnant women in Đakovština, Croatia. The study was approved by the Ethics Board of the Faculty of Medicine, Josip Juraj Strossmayer University of Osijek. Each participant was informed about the research and gave written consent. Inclusion criteria were: healthy pregnant women (without mental and somatic disorders), pregnancy without complications, and pregnant women without mood disorders during previous pregnancies (data obtained from gynecologist). Exclusion criteria were: mood disorders in previous pregnancies and after delivery, the use of psychopharmaceutics, complications during pregnancy, preeclampsia, eclampsia, and gestational diabetes.

The selected cohort group of women was prospectively monitored, and their psychological condition was evaluated at three time-points: in the last trimester of pregnancy (36th week of pregnancy), during the first home visit after leaving the maternity ward (mean infant age 7.7 days), and at the end of the third month after birth (90 days) by the visiting nurse at the participants’ homes. Out of 209 participants from the first time-point, 194 withdrew at the second time-point, and 160 at the third time-point. The most common reasons for withdrawal from the study was moving to another city or the participation of Roma women who changed residence or were no longer allowed to participate in the study.

### 2.2. Measures

The socio-demographic questionnaire adjusted for pregnant women consisted of 23 questions regarding gender, age, marital status, employment, education, childbirth, previous pregnancies, current pregnancy, and habits. The puerperea-adjusted clinical questionnaire consisted of 11 questions about general characteristics, i.e., childbirth, decision about breastfeeding, length of breastfeeding, breastfeeding support, and five questions for nurses about breastfeeding and baby progression assessment. Baby progression assessment was made by the same nurse and with the same pediatric measuring device for measuring weight, length, and head circumference in the first week and at the end of the third month.

The Edinburgh Postnatal Depression Scale (EPDS) is a self-report scale used for the assessment of the common symptoms of depression in women after birth. It was developed by Cox et al., (1987) [21], validated for pregnancy by Bergink et al., (2011) [22], and it is used broadly for identification of women at risk of perinatal depression during or one year after pregnancy. The Croatian version was validated by Nakić et al., (2013) [23]. The questionnaire contains ten items about cognitive and behavioral symptoms of depression, and each item is scored on a 4-point scale (0–3) depending on the severity of symptoms. The minimum and maximum total score range is 0–30, with a higher score indicating a higher degree of depression, and a score of 10 taken as a cutoff score.

The Beck Depression Inventory (BDI) is a 21-item self-reporting questionnaire regarding symptoms of depression, based on the Diagnostic and Statistical Manual of Mental Disorders (DSM-IV) [24]. It is used for evaluating the severity of depression in healthy and psychiatric populations. The inventory consists of 21 items with multiple response options presented on a scale of 0 to 3, depending on the severity of symptoms within two weeks prior to the administration of the questionnaire. The minimum and maximum score range from 0 to 63, with higher scores indicating greater symptom severity. Scores of 0–13 indicate minimal severity, 14–19 mild severity, 20–28 moderate severity, and 29–63 severe depression. The BDI has been standardized for the Croatian language [25].

The Beck Anxiety Inventory (BAI) is a 21-item self-reporting questionnaire constructed to measure somatic symptoms of anxiety and distinguish between anxiety and depression [26]. Respondents indicate how much they have been bothered by each symptom over the past week. Responses are rated on a 4-point Likert scale and range from 0 (not at all) to 3 (severely). The total score ranges from 0–63 indicating the overall feeling of anxiety, with a higher score meaning a higher intensity of anxiety. Depending on the total score, results can be 0–7 (normal or no anxiety), 8–15 (mild to moderate anxiety), 16–25 (moderate to severe anxiety), and 26–63 (severe anxiety). The inventory is simple and used broadly in daily clinical practice; it has been used in Croatia [27].

### 2.3. Statistical Analysis

Data are presented as absolute frequencies, means with standard deviations, and medians with interquartile range (IQ range), where appropriate. The normality of the distribution of the observed numeric variables was tested using the Kolmogorov–Smirnov test. Correlation between variables was assessed with the Spearman’s rank-order correlation coefficient. Differences between independent samples were tested using the standard inferential statistical tests employed with two independent samples: Student’s *t*-test. The level *p* < 0.05 was chosen for the statistical significance assessment of the obtained results. The analysis was conducted using the SPSS software (ver. 16.0, SPSS Inc., Chicago, IL, USA).

## 3. Results

The age of participants ranged from 18 to 46 years, with average age M = 28.04 years (SD = 5.18). Almost all participants were married (96.7%), more than half (53.1%) live with close family (spouse, child/children). A third of them live in a place with less than 1000 inhabitants (34.5%). Most of the participants had completed high school (67.5%).

The internal consistency of the used scales was determined for the first, second, and third time-point (Table 1).

On the EPDS scale of perinatal depression, 85.7% of participants had a score of less than 10, which indicates that they were not at risk of PPD. The results measured by the BDI showed that 94.1% of participants fall in the score indicating minimal depression. On the BIA scale, 74% of participants were without anxiety, and 18.2% had mild anxiety.

The research showed that 173 (89.2%) mothers breastfed their child after they had left the hospital. At the end of the third month, this percentage decreased to 62.5%. If we look at the measures of central tendency (mean, median, and dominant value), it can be noted that the values of the physical characteristics of children are approximate in both time-points, i.e., first week and three months after birth.

The marginal level of statistical significance indicates a potentially higher growth, i.e., a greater change in length of children who were breastfed three months after the birth than children who were not breastfed three months after birth (*t* = 1.968, *p* = 0.05). According to the results, changes in head circumference are not dependent on breastfeeding in the third month (Table 2).

The results (χ^2^ = 4.746, *p* < 0.05) show that postpartum mothers with low risk of PPD breastfed their children more often than mothers at mild or severe risk of perinatal depression. A significantly higher percentage of mothers from the latter group did not breastfeed their children at the end of the third month (Table 3).

The relationship between the presence of breastfeeding and depression and anxiety three months after giving birth is somewhat different (Table 4). Differences in depression were found three months after giving birth between mothers who breastfed and those who did not (*t* = 2.05, *p* < 0.05). The results show that mean values on the BDI scale three months after giving birth were higher in mothers who did not breastfeed (M = 3.53) than those who did (M = 2.28). Postpartum anxiety measured by the BAI was statistically negatively correlated (rs-, 430) with the duration of breastfeeding.

## 4. Discussion

The American Academy of Pediatrics’ Policy on Breastfeeding highlights that proper nutrition starts with breastfeeding exclusively for about six months of life. According to data from the Croatian Health and Statistics Yearbook for 2013, 71.8% of infants up to two months of age were exclusively breastfed, while 14.4% were breastfed with the addition of breast-milk substitutes. At three to five months of the infant’s age, the rate of exclusively breastfed infants decreased to 58.2%, and 20.1% of them were breastfed with the addition of breast-milk substitutes. After six months, only 19% of infants were exclusively breastfed [28]. The results in our study are somewhat above the Croatian average, with 89.2% of mothers breastfeeding their child after leaving the hospital and 62.5% breastfeeding at the end of the third month. Still, compared to UNICEF data from 2018, Croatia has not reached the global average percentage of infants exclusively breastfed in the first five months of life.

There are six critical periods in which to make breastfeeding decisions: pregnancy, the first 24 h after birth, 1 to 2 weeks after birth, 6 to 8 weeks after birth, 4 to 6 months after birth, and 12 months after birth [29]. This is the time when most breastfeeding problems are expected and when mothers give up breastfeeding more quickly. Breastfeeding performance is influenced by a woman’s attitudes and beliefs about her breastfeeding performance, that is, breastfeeding self-efficacy [30,31]. Self-efficacy determines whether the mother will choose to breastfeed, how persistent she will be, and how she will respond emotionally to the difficulty of breastfeeding. Breastfeeding self-efficacy is affected by previous breastfeeding experience, indirect experience, verbal persuading about breastfeeding, emotional excitement, pain, fatigue, stress response, and anxiety. An important indicator of breastfeeding success is the normal progression of the baby, which is also a factor in encouraging the mother to continue breastfeeding.

Breastfeeding is associated with the well-being of both mother and child. It develops the emotional connection between mother and child, builds feelings of safety and protection, and as such, is important for the cognitive and emotional development of the child [32]. The connection between breastfeeding and PPD is bidirectional; not engaging in breastfeeding may increase the risk of PPD, but also PPD may lead to lower rates and early cessation of breastfeeding [33]. A study conducted in the Republic of Croatia on pregnant women in the third trimester of pregnancy showed that 30.3% had symptoms of PPD [34]. In our study, about 15% of participants reached a score of more than nine on the EPDS scale, indicating a risk for PPD development. Additionally, mean values on the BDI scale three months after giving birth were higher in mothers who did not breastfeed their child (M = 3.53) than those who did. Our findings are in line with numerous studies reporting higher levels of depressive symptoms in women who are not breastfeeding [16]. In a study by Nishioka et al., it was found that the proportion of women with a score on the EPDS scale higher than 9 is much lower among women who breastfeed [35].

Although PPD is considered a risk factor for breastfeeding cessation, previous negative breastfeeding experiences can be a risk factor for PPD [36]. Postpartum depression might cause adverse effects on maternal self-esteem and cognition. Women with depressive symptoms may have more inadequate interactions with their newborns, which, in turn, increases the risk of breastfeeding difficulties. Breastfeeding mothers had better sleep patterns and an average of 40 to 45 min longer sleep. They also experienced less fatigue, which affected their symptoms of depression [37]. Mothers who were better able to breastfeed from the beginning and interact with their infants were more satisfied [38]. On the other hand, depressed mothers experienced significant breastfeeding problems and felt unsatisfied with breastfeeding, which has been reported as a common concern for mothers with PPD symptoms [39]. The mechanisms of interaction between PPD and breastfeeding are multifactorial, with depression influencing mothers’ self-esteem and relationship with infants.

To the best of authors’ knowledge, this is the first study on the subject of the protective effect of breastfeeding on postpartum depression in Croatia. The results of the study could serve to develop new strategies, preventive methods, and approaches for women in this sensitive period. The limitation of this study was the withdrawal of some participants before completion, which could potentially lead to distorted results. Unfortunately, the statistical analyses only included group comparisons without controlling for potential covariates. Therefore, it was not possible to identify the cause of the results or conclusively determine the directional effects. Additionally, the research could be strengthened by conducting a longitudinal study and following women six months and one year after birth.

## 5. Conclusions

Depression is a common complication of childbirth. Nonbreastfeeding mothers are more depressed and anxious compared to breastfeeding mothers. This study implies a need for early recognition and intervention in this vulnerable group of women and infants.

## Figures and Tables

**Table 1:** Estimation of the reliability of a psychometric test.

	Cronbach’s Alpha (α)		
Scale	Pregnancy	First Week After Birth	Three Months After Birth
Edinburgh Postnatal Depression Scale	0.837	0.853	0.784
Beck’s Depression Inventory	0.799	0.819	0.812
Beck’s Anxiety Inventory	0.887	0.915	0.917

**Table 2:** The testing difference in physical characteristics between children who were breastfed and those who were not breastfed three months after birth.

Change in Physical Characteristics	Breastfed	*N*	Mean	Standard Deviation	*t*	Degrees of Freedom (df)	*p*
Weight (g)	Yes	100	2714.24	639.06	1.734	157	0.085
	No	59	2533.19	630.59			
Length (cm)	Yes	100	11.57	3.10	1.968	157	0.051
	No	59	10.46	3.96			
Head circumference (cm)	Yes	91	4.68	1.87	0.538	145	0.591
	No	56	4.52	1.64			

*t*-test.

**Table 3:** Breastfeeding three months after birth in mothers with low, mild, or high risk for perinatal depression.

		Breastfeeding	Nonbreastfeeding	Total	χ^2^ (df = 2)	*p*
Low risk	*N*	83	40	123	4.746	0.029
	%	67.5%	32.5%	100%		
Mild or high risk	*N*	17	20	37		
	%	45.9%	54.1%	100%		
Total	*N*	100	60	160		
	%	62.5%	37.5%	100%		

Testing the difference (Chi-square).

**Table 4:** Testing the difference in depression and anxiety during pregnancy, after birth, and three months after birth in mothers who breastfed and those who did not three months after birth.

	Breast Feeding	*N*	Arithmetic Mean	Standard Deviation	*t*	Degrees of Freedom (df)	*p*
After birth	Yes	98	3.21	0.37	−0.929	152	0.354
	No	56	3.86	0.64			
Three months after birth	Yes	99	2.28	0.31	−2.050	157	0.042
	No	60	3.53	0.59			
After birth	Yes	99	4.08	0.52	−1.276	155	0.204
	No	58	5.40	1.02			
Three months after birth	Yes	100	3.24	0.52	−1.911	157	0.058
	No	59	5.14	0.95			

*t*-test.
